# An Unusual Presentation of Succinic Semialdehyde Dehydrogenase Deficiency: A Fatal Case of Severe Progressive Seizures in a Four-Month-Old Infant

**DOI:** 10.7759/cureus.58326

**Published:** 2024-04-15

**Authors:** Sara Idkaidak, Lila H Abu-Hilal, Duha I Barghouthi, Osama Atawneh, Abdelrazzaq Abumayaleh, Firas Alqarajeh

**Affiliations:** 1 Pediatric Medicine, Faculty of Medicine, Al-Quds University, Jerusalem, PSE; 2 General Practice, Al-Quds University, Jerusalem, PSE; 3 Faculty of Medicine, Al-Quds University, Jerusalem, PSE; 4 Pediatrics, Pediatric Medicine, Palestine Red Crescent Society (PRCS) Hospital, Hebron, PSE; 5 Pediatrics, Palestine Red Crescent Society (PRCS) Hospital, Hebron, PSE

**Keywords:** fatal outcome, pediatric seizure, gamma-aminobutyric acid (gaba), rare genetic diseases, ssadh

## Abstract

Succinic semialdehyde dehydrogenase (SSADH) deficiency is a rare genetic condition with approximately 450 patients reported worldwide, inherited in an autosomal recessive manner affecting gamma-aminobutyric acid (GABA) metabolism, characterized by varied clinical features. We report a fetal case of a four-month-old female infant presenting with severe, progressive seizures leading to fatality. Despite aggressive medical interventions, including multiple antiepileptic medications and a ketogenic diet, the patient's condition deteriorated rapidly. Genetic testing revealed a homozygous mutation in the aldehyde dehydrogenase 5 family member A1 (ALDH5A1) gene. This present case emphasizes the difficulties in controlling SSADH deficiency and emphasizes the necessity for additional studies on successful therapy approaches.

## Introduction

Succinic semialdehyde dehydrogenase (SSADH) deficiency is an uncommon genetic condition characterized by disrupting the normal processing of gamma-aminobutyric acid (GABA), a crucial inhibitory neurotransmitter in the brain. This condition follows an autosomal recessive pattern of inheritance [1]. The condition is caused by mutations in the *ALDH5A1 *gene, which is a member of the aldehyde dehydrogenase 5 family located on chromosome 6p22. Although the pathophysiology of this disease is not fully understood, part of the mechanism of the disease is related to the abnormal accumulation of the compound succinic semialdehyde caused by SSADH deficiency, which in turn is converted to gamma-hydroxybutyric acid (GHB)[2]. Also, the impairment of the function of the SSADH enzyme leads to a disruption of the metabolism of GABA. Both GABA and GHB contribute to pathophysiology.

The clinical features of SSADH deficiency are widely variable. SSADH deficiency can present at various ages, including late infancy and early childhood. The clinical presentation is marked by developmental delay and intellectual disability, along with notable delays in expressive language, low muscle tone, lack of coordination, movement abnormalities, and seizures [3,4]. Seizures can be seen in this disease and usually, the patient presents with generalized seizures and can be managed with appropriate medications.

Here we describe an unusual presentation of a four-month-old female patient diagnosed with SSADH deficiency who presented with severe progressive seizure leading to the death of the patient.

## Case presentation

A full-term, four-month-old female infant was delivered via normal vaginal delivery. Her 35-year-old mother, a healthy gravida seven para seven woman, experienced an uneventful pregnancy. The baby’s birth parameters were within normal ranges with a birth weight of 2500 g. Her parents were first-degree cousins. She also had a sibling who died at the age of one year due severe convulsion that was difficult to manage. She died at our hospital of respiratory failure after a prolonged convulsion requiring many antiepileptic drugs. Genetic testing was ordered for her at that time but unfortunately was not done due to financial reasons.

The patient was well till the age of one month when she started to have recurrent abnormal movement in the form of generalized tonic-clonic movement in both upper and lower limbs associated with uprolling of eyes that lasted for a few minutes so she was admitted to a hospital for investigations. An electroencephalogram (EEG) showed bilateral epileptogenic activity, then she was started on levetiracetam. After that abnormal movement resolved and the patient was discharged home.

**Figure 1:**
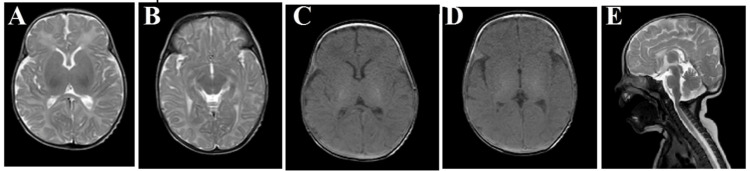
Brain MRI (A,B) (T2-weighted) showing a coronal view of the brain. (C,D) (T1-weighted) showing a coronal view of the brain. E (T2-weighted) showing a sagittal view of the brain.

At the age of two months, abnormal movement recurred and sodium valproate was added. Brain magnetic resonance imaging (MRI) was done at that time with normal cortex, white matter, cerebellum, and ventricular system. The brain showed normal myelination adequate for age (Figure 1).

At the age of four months, abnormal movement recurred again but in a much more frequent manner. The family sought medical advice at our hospital and the patient was admitted.

**Figure 2:**
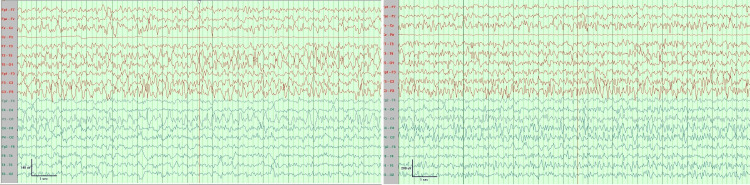
EEG monitoring EEG showed predominantly runs of high-voltage sharp wave spikes, slow pikes waves, and episodes of prolonged electrographic seizure (left side initiation that became generalized).

**Figure 3:**
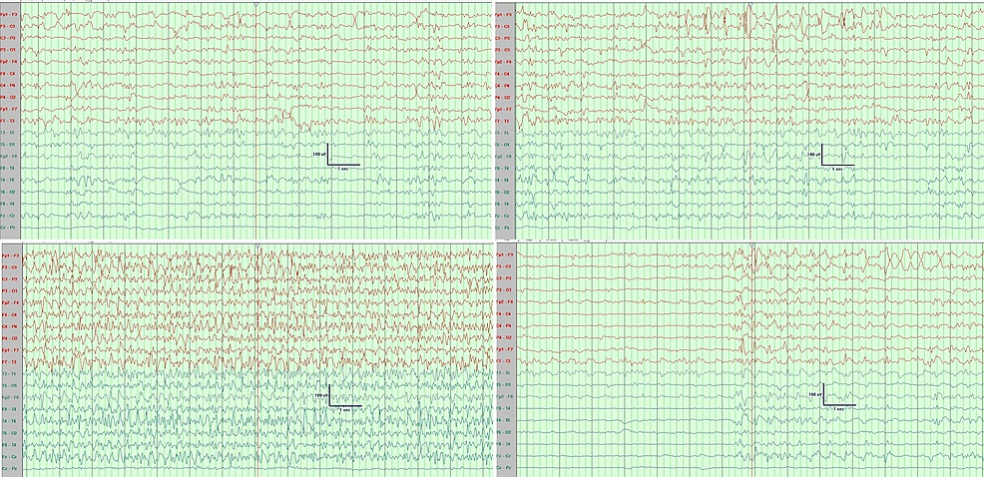
EEG monitoring EEG showing low-voltage background activity with frequent bilateral short runs of sharp waves, slow waves, spikes and polyspikes frequently synchronized. In addition to periods of bilateral nearly continuous runs of high-voltage sharp waves, slow spike-wave (SSW) activities for variable duration (generalized seizures).

On admission, she had focal dyscognitive seizure in the form of facial twitching, eye blinking, and myoclonic jerky movement of extremities alternating between the left and right side. She was given a bolus infusion of phenytoin and connected to EEG monitoring which showed predominantly runs of high-voltage sharp wave spikes, slow pikes waves, and episodes of prolonged electrographic seizure (left side initiation that became generalized) (Figure 2). She was in status epilepticus for which she was transferred to the pediatric intensive care unit and was intubated. She was given a midazolam infusion escalating the dose up to 8 mg/kg/minute without complete control. The next day thiopental was added up to 5 mg/kg/hour. After thiopental, there was a partial response with decreased frequency of clinical seizure but EEG did not show significant improvement (Figure 3). In the last few days, she was on levetiracetam, sodium valproate, clobazam, topiramate, phenytoin, and the ketogenic diet. Unfortunately, there was no improvement in her condition and she continued to have seizures. On the last day of life, she was extubated and connected to a noninvasive mechanical ventilator. After extubation, she was encephalopathic. A few hours after extubation, she developed respiratory distress with O_2_ desaturation. So, she was intubated again and despite all the anticonvulsant medication, there was no response. She then developed respiratory failure and her condition deteriorated. She suddenly developed bradycardia and despite resuscitation, she died. Before her death, a blood sample was saved for genetic testing, and when the family agreed to do the genetic analysis.

Whole exome sequencing was done from the blood sample. It showed a homozygous mutation G->A substitution at chr6:24505155. Akaboshi et al. predicted this mutation to cause an abnormal protein translation of the ALDH5A1 protein at amino acid position 223 [5].

The sequencing utilized the IDT xGen Exome Research Panel v2.0 for protein-coding regions and the xGen Human mtDNA Research Panel v1.0 for mitochondrial DNA on the Illumina NOVASEQ 6000 platform, adhering to stringent European Molecular Genetics Quality Network (EMQN) standards. Variants were carefully filtered, excluding intronic variants >8 base pairs from splice sites, synonymous variants >3 base pairs from splice sites, and variants with >1% minor allele frequencies in the GnomAD database.

## Discussion

SSADH deficiency (SSADH-D; OMIM #271980) is an autosomal-recessively inherited disorder affecting GABA degradation due to mutations in the *ALDH5A1* gene on chromosome 6p22.3 [1]. The first documented case of SSADH-D, identified in 1981, revealed GHB excretion in urine, with subsequent confirmation of SSADH enzyme deficiency two years later [3,6].

The clinical presentation of SSADH-D is remarkably diverse, often leading to diagnostic challenges, particularly in cases without familial precedent [3]. Manifestations encompass global developmental delay, hypotonia, epilepsy, extrapyramidal symptoms, and hyporeflexia [1].

Notably, SSADH-D commonly exhibits intellectual disability and mental health conditions, autism-like traits, impaired speech, and sleep disturbances [1]. Nearly all patients experience developmental delay and intellectual disability, while approximately 80% contend with ataxia and muscular hypotonia. Epileptic seizures emerge in the majority, commencing in late childhood and persisting into adulthood, varying from absence seizures to generalized forms [3].

In the context of this discussion, our presented case adds a fetal presentation to the spectrum of SSADH-D manifestations. The four-month-old female infant demonstrated an unusual and severe clinical course, marked by recurrent seizures resistant to conventional antiepileptic therapies, ultimately leading to a fatal outcome. This case underscores the heterogeneity of SSADH-D and emphasizes the critical need for heightened awareness and early diagnosis, especially in the absence of a familial history. Horino et al. described a nine-month-old patient with severe presentation and status epileptics that required ketamine infusion to manage which was not used in our case [7]. EEG findings were similar to our case including sharp waves with LT side initiation. This patient survived but had rapid regression, refractory myoclonus, and severe progressive brain atrophy later on.

Preclinical studies using aldehyde dehydrogenase 5a1−/− (aldh5a1−/−) mice, a murine SSADH-D model, suggest the potential of enzyme replacement therapy. Neuronal stem cells derived from these mice have proven valuable for therapeutic investigations, revealing down-regulated GABAA receptor transcripts, indicating the potential therapeutic relevance of agents modulating chloride channel activity. Recent therapeutic prospects involve mTOR inhibitors, emphasizing the evolving landscape of SSADH-D treatment possibilities [2].

While our patient's journey ended in tragedy, the lessons learned from her case may guide future research and clinical efforts aimed at improving the understanding and management of SSADH-D. Continued exploration of novel therapeutic trials, informed by insights gained from individual cases, remains crucial in the pursuit of more effective interventions and improved outcomes for individuals affected by this rare and challenging disorder.

## Conclusions

In conclusion, our case report demonstrates the challenging and often devastating clinical course of SSADH-D. The four-month-old female infant presented with an atypical and severe manifestation of the disorder, marked by relentless seizures that proved refractory to standard antiepileptic interventions. Despite intensive medical efforts, the patient's condition deteriorated rapidly, ultimately leading to a tragic outcome. This case underscores the urgent need for increased awareness and early diagnosis of SSADH-D which can be achieved through genetic testing, especially with previous family history. Future treatment options should be investigated in such severe cases.
